# Comparative Physiological and Proteomic Analyses of Poplar (*Populus yunnanensis*) Plantlets Exposed to High Temperature and Drought

**DOI:** 10.1371/journal.pone.0107605

**Published:** 2014-09-16

**Authors:** Xiong Li, Yunqiang Yang, Xudong Sun, Huaming Lin, Jinhui Chen, Jian Ren, Xiangyang Hu, Yongping Yang

**Affiliations:** 1 Key Laboratory for Plant Biodiversity and Biogeography of East Asia, Kunming Institute of Botany, Chinese Academy of Sciences, Kunming, China; 2 Plant Germplasm and Genomics Center, The Germplasm Bank of Wild Species, Kunming Institute of Botany, Chinese Academy of Sciences, Kunming, China; 3 University of Chinese Academy of Sciences, Beijing, China; 4 Key Laboratory of Forest Genetics & Biotechnology, Nanjing Forestry University, Nanjing, China; 5 Department of Grassland Science, Yunnan Agricultural University, Kunming, China; Institute of Genetics and Developmental Biology, Chinese Academy of Sciences, China

## Abstract

Plantlets of *Populus yunnanensis* Dode were examined in a greenhouse for 48 h to analyze their physiological and proteomic responses to sustained heat, drought, and combined heat and drought. Compared with the application of a single stress, simultaneous treatment with both stresses damaged the plantlets more heavily. The plantlets experienced two apparent response stages under sustained heat and drought. During the first stage, malondialdehyde and reactive oxygen species (ROS) contents were induced by heat, but many protective substances, including antioxidant enzymes, proline, abscisic acid (ABA), dehydrin, and small heat shock proteins (sHSPs), were also stimulated. The plants thus actively defended themselves against stress and exhibited few pathological morphological features, most likely because a new cellular homeostasis was established through the collaborative operation of physiological and proteomic responses. During the second stage, ROS homeostasis was overwhelmed by substantial ROS production and a sharp decline in antioxidant enzyme activities, while the synthesis of some protective elements, such as proline and ABA, was suppressed. As a result, photosynthetic levels in *P. yunnanensis* decreased sharply and buds began to die, despite continued accumulation of sHSPs and dehydrin. This study supplies important information about the effects of extreme abiotic environments on woody plants.

## Introduction

Global warming, the most typical manifestation of worldwide climate change, is a focus of increasing attention. Although warming experiments have often been used to simulate future climate conditions, this approach is limited by the unproven assumption that plant responses to experimental warming match their long-term responses to global warming [1]. Within natural habitats, however, plants are often subjected to a combination of different abiotic stresses, each with the potential to exacerbate the damage caused by the others. Recent studies have provided evidence that the molecular, biochemical, and physiological responses of plants to a combination of abiotic stresses are unique and cannot be directly extrapolated from their responses to each stress applied separately [2]. Because high temperatures can increase evapotranspiration rates [3], warming is usually accompanied by drought; plant growth is thus limited directly by heat stress or indirectly via water shortage. In fact, drought and heat shock are common stress factors that often reduce crop yield by more than 50% [4]. They are also two of the most important abiotic stress factors impacting the natural distributions of woody plants and limiting global ecosystem production [5].

Research on plant responses to heat, drought, or their combination has mainly focused on model plants and crops that are herbaceous, such as wheat [4], [6]–[9], sorghum [7], potato [10], pea [11], bean [12], [13], and tobacco [14]. The effects of high temperature and drought on the growth and development of woody plants have rarely been studied, and little is known regarding how the combination of these two factors impacts woody plants.

Yunnan poplar (*Populus yunnanensis* Dode), native to high altitude areas of southwestern China, is one of the woody plants most commonly used in stress resistance studies [15]. This plant plays an important role in forestry production, afforestation, and environmental conservation because of its fast growth rate, high biomass, and large populations [16]. Because *P. yunnanensis* populations have recently experienced climate warming and continuous drought stress in southwestern China, an understanding of the combined effects of heat and drought on this species is a research priority. Previous studies of *P. yunnanensis* have involved greenhouse experiments to determine the effects of abiotic stresses on its growth and physiology. The applied stresses have included heavy metals, salinity, acid rain, elevated CO_2_, warming, drought, UV-B, and their various combinations [16]–[23], but the combined effects of heat and drought on *P. yunnanensis* are still largely unknown. In the present study, we performed experiments to explore the response of *P. yunnanensis* to sustained high temperature and drought using comparative proteomic and physiological analyses. To precisely determine the effect of combined heat and drought stress, we also performed comparison experiments involving the application of separate high temperature and drought treatments. We aimed to understand how global climate change may affect woody plants, with *P. yunnanensis* used as a model.

## Results

### Changes in phenotype and physiological status

**Figure 1:**
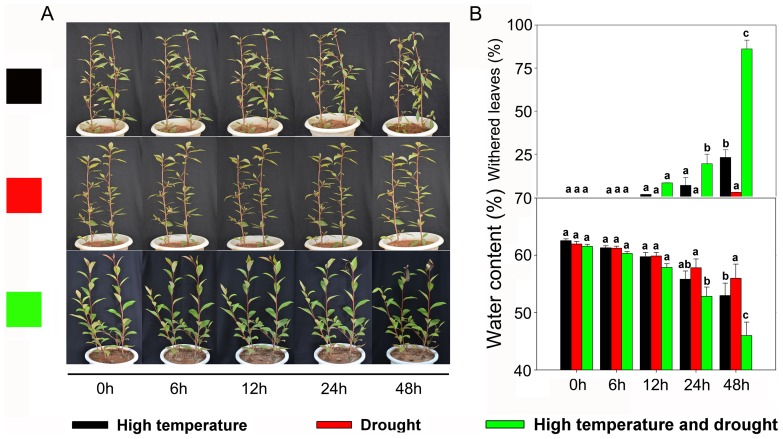
Effects of sustained heat, drought, and combined heat and drought on the morphology and relative water content of leaves of *Populus yunnanensis* plantlets. (**A**) Changes in plantlet morphology. (**B**) Number of withered leaves on plantlets. Data represent the means of five replicate experiments (± SE). Means labeled with different letters were significantly different according to Tukey's test (*P*<0.05). (**C**) Changes in plantlet leaf water content. Data represent the means of five replicate experiments (± SE). Means labeled with different letters were significantly different according to Tukey's test (*P*<0.05).

*Populus yunnanensis* plantlets exhibited various phenotypes under different treatments. When plantlets were exposed to either high temperature or drought, a weak morphological change was detected throughout the 48-h stress (Figure 1A). During early stages (0–12 h) of combined heat and drought stress, no significant changes were observed in morphology. However, the buds of *P. yunnanensis* exhibited apparent withering by 24 h, which was even more pronounced at 48 h (Figure 1A). The number of withered leaves and leaf water content displayed little change over 48 h of exposure to high temperature stress (Figure 1B), and remained relatively stable under single drought stress (Figure 1B). When exposed to a combination of high temperature and drought, treated plants had a greater number of withered leaves and slightly decreased leaf water content from 0 h to 24 h compared with the controls (0 h), with both of these parameters changing drastically after 24 h (Figure 1B).

**Figure 2:**
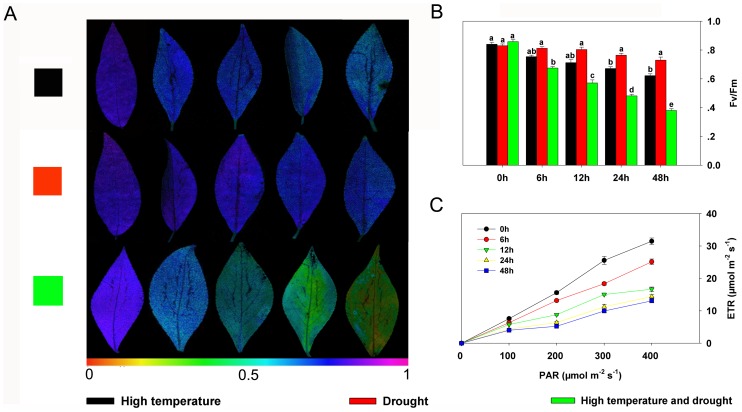
Effects of different treatment durations on leaf photosynthesis in *Populus yunnanensis* plantlets under different stresses. (**A**) *F_v_/F_m_* images (bottom). The pseudocolor code depicted at the bottom of the image ranges from 0 (red) to 1.0 (purple). The experiment was replicated three times with similar results. One representative result is shown. (**B**) Average *F_v_/F_m_* values. *F_v_/F_m_* was determined for whole leaves exposed to different treatments. Data represent the means of five replicate experiments (± SE). Means labeled with different letters were significantly different according to Tukey's test (*P*<0.05). (**C**) Electron transport rates determined after different durations of exposure to heat and drought stress. The data represent the means of five replicate experiments (± SE).

Maximum quantum yield (the ratio of variable to maximum fluorescence; *F*_v_ */F*_m_) and electron transport rates (ETRs) of photosystem II (PSII), which can indicate plant photosynthetic capacity [24], also showed various changes under different stresses. The ratio of *F*_v_ to *F*_m_ of plantlets exposed to high temperature changed significantly after 24 h of stress (Figure 2A and B), but was only slightly changed in plantlets exposed only to drought (Figure 2A and B). In contrast, *F*_v_/*F*_m_ and ETR both decreased significantly in plantlets treated to 40°C without watering (Figure 2). More specifically, *F*_v_ */F*_m_ values of samples treated for 6, 12, 24, and 48 h were 16.5, 29.3, 40.4, and 53.0% lower, respectively, compared with the controls (0 h) (Figure 2A and B), and the respective ETRs of these treated samples were 20.1, 38.9, 53.0, and 58.2% lower than the controls (Figure 2C).

### Changes in proline, malondialdehyde (MDA), and reactive oxygen species (ROS) contents

**Figure 3:**
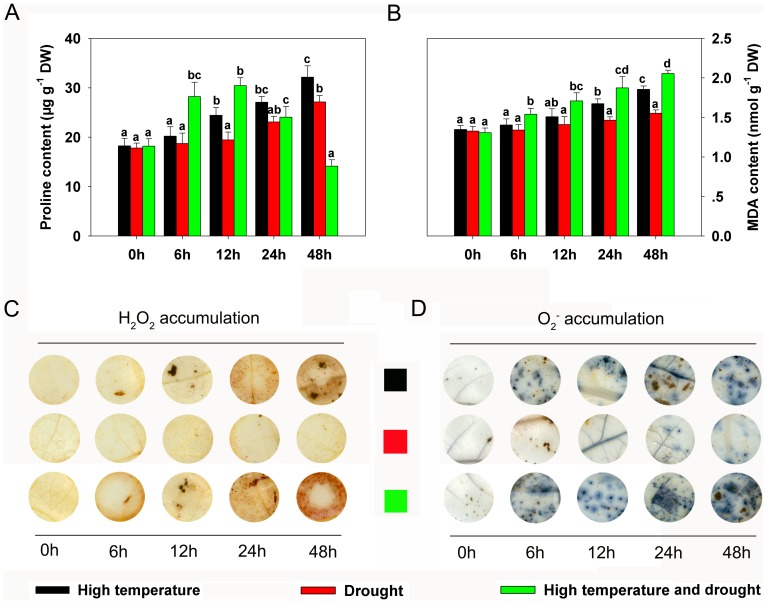
Accumulation of proline, malondialdehyde (MDA), and reactive oxygen species (ROS) (H_2_O_2_ and O_2_^−^) in *Populus yunnanensis* plantlets after different durations of exposure to different stresses. (**A**) Proline content at different times under heat, drought, or combined heat and drought. (**B**) MDA content at different times under heat, drought, or combined heat and drought. Data (B and C) represent the means of five replicate experiments (± SE). Means labeled by different letters were significantly different according to Tukey's test (*P*<0.05). (**C**) *In situ* detection of changes in leaf H_2_O_2_ levels at different times under heat, drought, or their combination. (**D**) *In situ* detection of changes in leaf O_2_^−^ levels at different times under heat, drought, or combined heat and drought.

Proline content is an important indicator of plant response to abiotic stress, especially drought. The proline content of *P. yunnanensis* rose gradually over time during individual heat or drought stress treatments (Figure 3A). Under combined heat and drought stress, proline content increased from 0 h to 12 h; after 24 h of stress, however, it decreased significantly (Figure 3A).

MDA and ROS such as H_2_O_2_ and O_2_^−^, which reflect grades of cellular oxidation [5], both gradually accumulated when plants were exposed to single or combined heat and drought stress treatments (Figure 3B–D). However, the degree to which MDA and ROS accumulated differed drastically under various treatments, with only slight accumulation under single drought stress and much greater accumulation under single heat stress (Figure 3B–D). When plants were stressed by heat and drought simultaneously, MDA and ROS were produced more rapidly and to greater degrees from 24 h to 48 h than from 0 h to 24 h (Figure 3B–D).

### Dynamics of antioxidant enzyme activities

**Figure 4:**
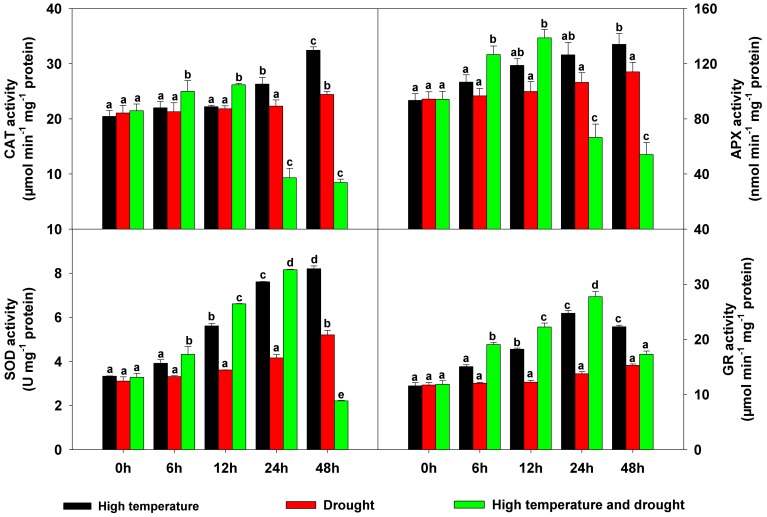
Changes in antioxidant enzyme activities in *Populus yunnanensis* plantlets after different durations of exposure to different stresses. The data represent the means of five replicate experiments (± SE). Means labeled with different letters were significantly different according to Tukey's test (*P*<0.05).

Activities of antioxidant enzymes, which play essential roles in maintaining ROS homeostasis in plants, were affected differently by heat, drought, and a combination of the two stresses (Figure 4). When exposed to either heat or drought stress, all four antioxidant enzyme activities rose by different degrees with increasing stress duration. Heat triggered greater increases in enzyme activities than did drought. Under the double-stress treatment, however, catalase (CAT) and ascorbate peroxidase (APX) activities were stimulated during the first 12 h and then displayed a significant decline from 24 to 48 h (Figure 4). In a similar fashion, superoxide dismutase (SOD) and glutathione reductase (GR) activities increased from 0 to 24 h, and then decreased markedly after 48-h stress (Figure 4).

### Protein profiling of the response of *P. yunnanensis* to different stresses

**Figure 5:**
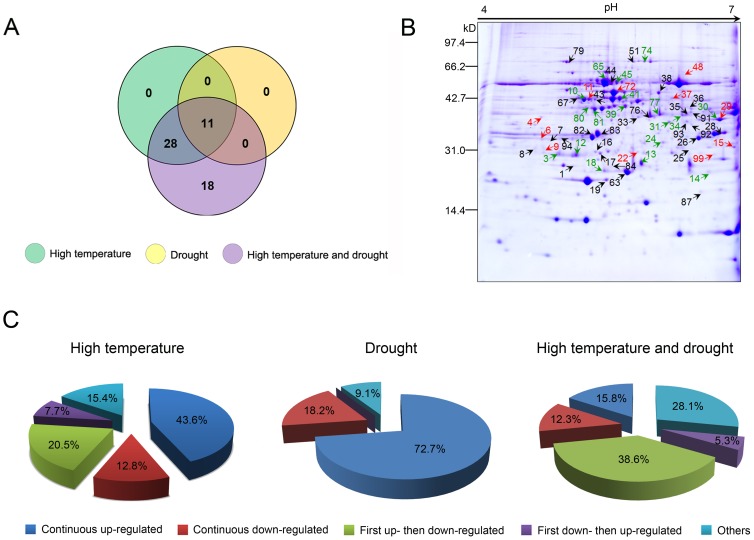
Results of comparative proteomics analyses of different treatments. **(A)** Representative 2-D gel showing spot numbers of identified proteins. Red spots represent common proteins differentially expressed under all three stresses. Green spots correspond to proteins specifically differentially expressed under combined stress. **(B)** Venn diagram of differentially expressed proteins under different treatments. **(C)** Expression patterns of differentially expressed proteins under different treatments.

To obtain a profile of proteins involved in *P. yunnanensis* stress response, we performed two-dimensional electrophoresis (2-DE) of samples subjected to high temperature, drought, and combined heat and drought stress. The 2-DE was repeated three times on each sample with similar results; therefore, one set of representative gels per treatment was visualized by Coomassie Brilliant Blue (CBB) staining (Figures S1, S2, and S3). After staining, more than 600 protein spots were detected within each treatment. Of these, we observed 47, 24, and 90 proteins whose expressions varied by at least 1.5-fold (*P*<0.05) among samples subjected to heat, drought, and combined heat and drought treatment, respectively. Fifty-seven of these differentially expressed proteins (Table S1) were unambiguously identified by matrix-assisted laser desorption/ionization-tandem time-of-flight mass spectrometry (MALDI-TOF-MS/MS) and screened against the NCBI nonredundant protein database (Table 1). Among these identified proteins, the expressions of 39, 11, and 57 were altered by high temperature, drought, and double-stress treatments, respectively (Figure 5A). Interestingly, the 11 proteins whose expressions were altered by drought stress were also affected by the other two treatments (Figure 5A and B), while the expressions of the 39 differentially expressed proteins observed under high temperature stress were also changed by combined heat and drought stress (Figure 5A). In addition, expressions of 28 proteins were altered by high temperature stress alone and in combination with drought (Figure 5A), while expressions of 18 proteins were changed only when subjected to combined heat and drought stress (Figure 5A and B). Under high temperature stress, 39 protein spots displayed five types of changes (17 continuously up-regulated, 5 continuously down-regulated, 8 first up- and then down-regulated, 3 first down- and then up-regulated, and 6 otherwise) (Figure 5C). Under drought stress, 11 protein spots exhibited three types of changes (8 continuously up-regulated, 2 continuously down-regulated, and 1 otherwise) (Figure 5C). Under combined high temperature and drought stress, 57 protein spots reflected five types of changes (9 continuously up-regulated, 7 continuously down-regulated, 22 first up- and then down-regulated, 3 first down- and then up-regulated, and 16 otherwise) (Figure 5C).

**Table 1:** Identification of differentially expressed proteins in leaves of *Populus yunnanensis* plantlets after different durations of heat and drought stress as analyzed by MALDI-TOF-MS/MS.

Spot	Protein name	Acc. No.^a^	Theo. *M*_w_/p*I* ^b^	Exp. *M*_w_/p*I* ^c^	SC^d^ (%)	Score^e^	Organism
**Defense related**							
3	desiccation-related protein LbLEA3_3–06	gi|169159964	21.12/6.93	21.98/4.88	29.5	40	*L. brevidens*
6	resistance protein	gi|37221893	18.35/5.21	26.84/4.59	57.1	39	*A. stenosperma*
12	Heat shock 22 kDa protein	gi|3122228	23.97/6.34	21.89/5.02	29.9	23	*G. max*
16	26.7 kDa heat shock protein	gi|122247294	26.71/6.78	22.62/5.27	26.7	23	*O. sativa*
18	Late embryogenesis abundant protein D-113	gi|126075	17.48/5.81	17.08/5.29	20.7	22	*G. hirsutum*
22	23.1kDa heat-shock protein	gi|147225064	23.24/5.04	22.06/5.79	28.0	40	*T. monococcum*
82	3-isopropylmalate dehydratase small subunit	gi|166989796	24.23/5.66	27.88/5.23	58.1	43	*A. sp.*
**Cell structure and division**							
7	small GTP-binding protein	gi|1053067	22.78/5.27	26.72/4.71	54.2	38	*S. lycopersicum*
30	Xyloglucan endotransglucosylase/hydrolase protein A	gi|38605156	34.15/6.99	35.87/6.69	30.8	34	*P. angularis*
48	Ectoderm-neural cortex protein 2	gi|81901549	66.87/6.24	63.79/6.33	23.4	40	*M. musculus*
51	Tetratricopeptide repeat protein 30A2	gi|81918137	76.87/5.08	78.95/5.72	20.7	33	*R. norvegicus*
63	Leucine-rich PPR motif-containing protein	gi|123910179	15.75/6.00	16.81/5.66	19.6	38	*X. tropicalis*
**Nucleic acid metabolism**							
8	retrotransposon protein, putative	gi|78708153	21.70/5.88	23.50/4.52	45.3	39	*O. sativa*
67	Arginine – tRNA ligase	gi|238688807	62.46/5.72	47.77/5.04	21.1	34	*T. sp*.
76	UPF0042 nucleotide-binding protein Sala_2050	gi|118574110	34.77/6.35	37.36/5.94	18.1	33	*S. alaskensis*
84	Single-stranded DNA-binding protein	gi|6647824	19.09/5.04	17.67/5.38	35.6	32	*T. pallidum*
87	Vigilin	gi|218511884	14.20/6.43	15.00/6.42	10.8	33	*H. sapiens*
**Redox metabolism**							
10	precursor of dehydrogenase dihydrolipoamide dehydrogenase 1	gi|224099079	54.48/7.24	46.92/5.10	25.4	39	*P. trichocarpa*
31	glyceraldehyde 3-phosphate dehydrogenase	gi|255537011	32.11/7.72	33.79/6.17	32.5	49	*R. communis*
33	Glyceraldehyde-3-phosphate dehydrogenase	gi|122222108	56.56/6.61	35.72/5.81	19.9	29	*O. sativa*
37	flavin-containing monooxygenase YUCCA	gi|171362744	46.08/9.08	44.06/6.33	19.5	39	*O. sativa*
39	Alternative oxidase 1c	gi|3913142	37.91/6.90	42.43/5.62	37.7	39	*A. thaliana*
80	Enoate reductase 1	gi|52788252	44.81/5.60	41.93/5.13	30.8	34	*K. lactis*
83	Dihydrodipicolinate reductase	gi|166224179	27.82/5.15	29.26/5.29	34.1	37	*S. sanguinis*
**Photosynthesis**							
13	plasma membrane H+-ATPase	gi|2605909	26.44/5.91	19.20/5.83	26.6	34	*K. virginica*
41	Oxygen-evolving enhancer protein 1	gi|131384	35.10/6.25	47.14/5.55	26.6	31	*P. sativum*
93	Phosphate import ATP-binding protein PstB	gi|123748310	31.21/6.25	34.38/6.33	57.2	42	*P. fluorescens*
**Signal transduction**							
15	Ras-related protein RABH1d	gi|75337262	23.21/6.38	24.13/6.84	41.0	26	*A. thaliana*
45	Peptide chain release factor 3	gi|122269173	59.32/5.17	59.74/5.51	29.7	39	*L. brevis*
79	Ribosome-releasing factor 2	gi|261277887	81.88/5.91	78.83/4.92	17.6	33	*D. persimilis*
**Energy metabolism**							
19	ribulose-1,5-bisphosphate/carboxylase large subunit	gi|313758185	18.35/5.24	15.33/5.40	30.5	67	*S. dodecandra*
24	ribulose-1,5-bisphosphate carboxylase/oxygenase large subunit	gi|17224644	27.83/6.21	25.5/6.1	36.5	80	*D. pyrenaica*
29	ribulose-1,5-bisphosphate carboxylase/oxygenase large subunit	gi|67079082	25.60/6.23	35.56/6.75	39.2	47	*D. villosa*
44	ATP synthase subunit beta, mitochondrial	gi|114421	59.93/5.95	60.79/5.40	32.3	46	*N. plumbaginifolia*
65	ATP synthase subunit beta	gi|190358701	53.73/5.09	59.33/5.35	45.3	59	*P. trichocarpa*
**Enzyme system**							
Antioxidant enzyme							
1	2-cys peroxiredoxin	gi|224140038	29.71/6.44	18.70/4.87	29.3	40	*P. trichocarpa*
9	putative ascorbate peroxidase APX5	gi|31980502	28.90/8.84	22.89/4.74	40.2	48	*A. thaliana*
14	catalase 2	gi|215959344	25.20/6.10	18.99/6.56	17.7	37	*V. unguiculata*
25	Glutathione S-transferase 16	gi|330250548	24.11/6.25	23.84/6.42	23.1	26	*A. thaliana*
28	Peroxidase 43	gi|26397928	35.81/5.68	29.2/6.74	12.4	25	*A. thaliana*
Synthase							
4	5-enol-pyruvylshikimate-phosphate synthase	gi|63334403	47.81/5.76	36.33/4.58	46.2	52	*C. sumatrensis*
36	S-adenosylmethionine synthase	gi|1346524	43.71/5.59	41.73/6.43	21.3	37	*P. deltoides*
38	Indole-3-glycerol phosphate synthase	gi|27735264	44.84/6.99	46.72/6.20	24.3	34	*A. thaliana*
91	Biotin synthase	gi|123725422	38.79/6.19	39.73/6.43	24.7	44	*S. glossinidius*
Kinase							
35	PTI1-like tyrosine-protein kinase At3g15890	gi|75335398	41.34/5.36	38.67/6.37	36.1	28	*A. thaliana*
74	Protein kinase C-like 1B	gi|42560537	81.24/6.67	78.28/5.85	15.8	40	*C. elegans*
81	Acetate kinase	gi|259709978	43.72/5.30	40.82/5.25	38.4	40	*C. botulinum*
92	Acetylglutamate kinase	gi|122279744	31.36/6.27	34.52/6.38	29.2	29	*L. borgpetersenii*
Phosphatase							
11	Probable protein phosphatase 2C 15	gi|75131368	48.62/5.72	46.92/5.17	30.1	23	*O. sativa*
26	Phytochrome-associated serine/threonine protein phosphatase 1	gi|75314041	35.38/4.93	27.26/6.53	23.8	30	*A. thaliana*
Transferase							
17	Caffeoyl-CoA O-methyltransferase	gi|3023419	28.01/5.02	22.06/5.32	32.8	25	*E. gunnii*
77	Acetyl-coenzyme A carboxylase carboxyl transferase subunit alpha	gi|254800799	36.62/5.79	38.23/6.05	30.7	40	*B. anthracis*
99	Octanoyltransferase	gi|171769182	25.08/6.62	21.45/6.71	23.4	33	*A. citrulli*
Mutase							
72	Phosphoglucosamine mutase	gi|166990410	49.20/5.41	50.96/5.48	26.0	34	*C. botulinum*
**Other proteins**							
34	UPF0496 protein At4g34320	gi|75213510	42.49/8.47	37.91/6.33	13.9	22	*A. thaliana*
43	predicted protein	gi|224109888	42.77/4.92	46.95/5.25	30.6	62	*P. trichocarpa*
94	UPF0135 protein CPE2004	gi|20978811	29.17/5.02	27.16/4.96	45.6	41	*C. perfringens*

a Database accession numbers according to NCBInr; **^b^**Theoretical *M*_w_/p*I*; **^c^**Experimental *M*_w_/p*I*; **^d^**Sequence coverage; **^e^**Mascot search score against the NCBInr database.

**Figure 6:**
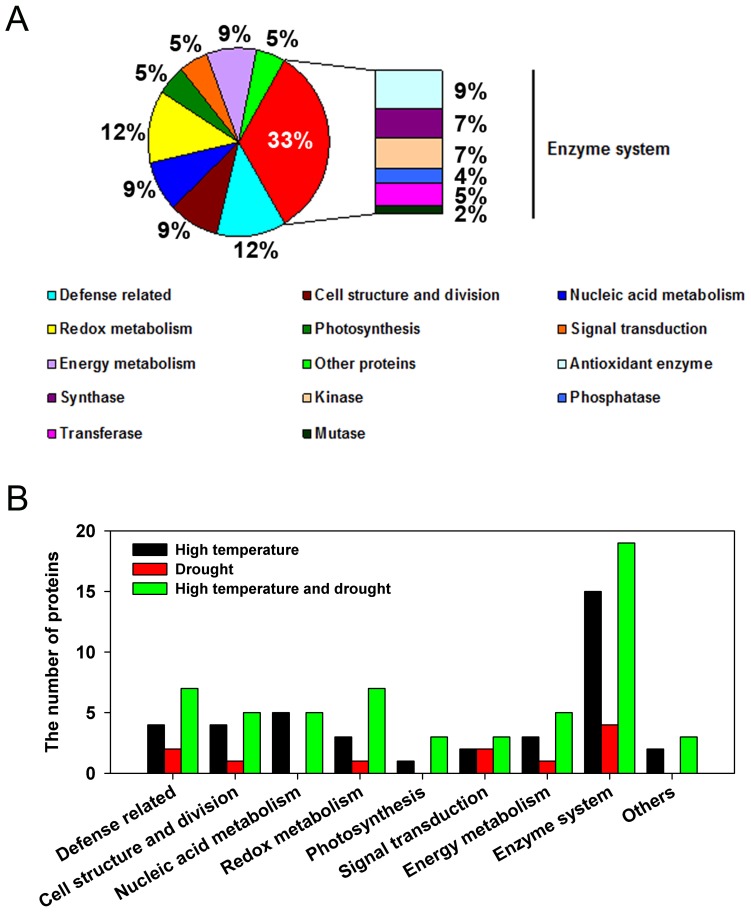
Functional classification of identified proteins and the number of proteins with various functions under different stresses. (**A**) Functional classification of the identified proteins based on NCBI annotation. (B) The number of proteins with various functions under different stresses.

The identified proteins could be classified into nine functional groups, namely, enzyme system (15, 4, and 19 under heat, drought, and combined stresses, respectively), defense-related (4, 2, and 7), cell structure and division (4, 1, and 5), nucleic acid metabolism (5, 0, and 5), redox metabolism (3, 1, and 7), photosynthesis (1, 0, and 3), signal transduction (2, 3, and 3), energy metabolism (3, 1, and 5), and other proteins (2, 0 and 3) (Figure 6), implying that these biological processes were affected by heat and drought stress. In particular, the enzyme system consisting of antioxidant enzyme (spots 1, 9, 14, 25, and 28), synthases (spots 4, 36, 38, and 91), kinases (spots 35, 74, 81, and 92), phosphatases (spots 11 and 26), transferases (spots 17, 77, and 99), and mutases (spot 72) accounted for nearly one-third (33%) of all differentially expressed proteins (Figure 6A). Moreover, proteins related to defense (spots 3, 6, 12, 16, 18, 22, and 82) and redox metabolism (10, 31, 33, 37, 39, 80, and 83) both constituted a large proportion of differentially expressed proteins, i.e., 12%, (Figure 6A), indicating their special roles during stress response. In addition, 18 proteins that were only differentially expressed under combined stress belonged to various functional categories with different expression patterns (Figures 5B and 6; Table 1), suggesting they were specifically induced or affected by combined stress but not by the individual stresses.

### Differing expressions of abiotic stress-related proteins

**Figure 7:**
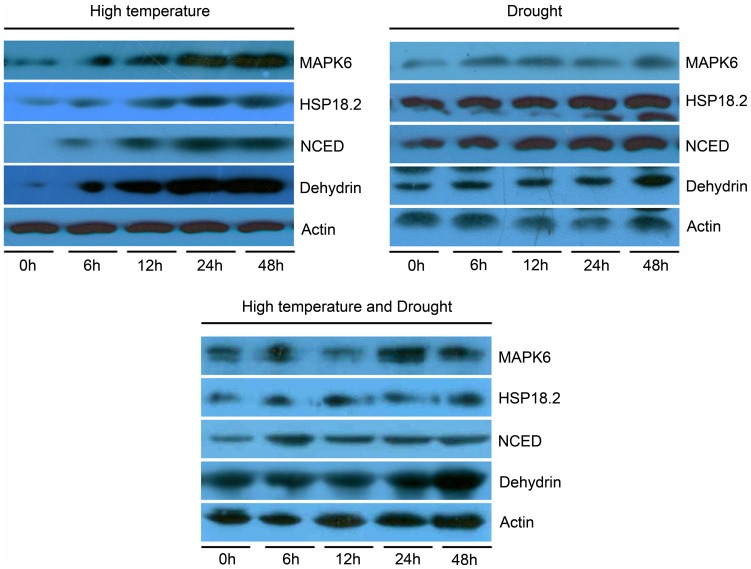
Western blot showing the effects of different stresses on plant mitogen-activated protein kinase 6 (MAPK6), heat shock protein 18.2 (HSP18.2), 9-cis-epoxycarotenoid dioxygenase (NCED), and dehydrin protein accumulation. Actin was included as a protein loading control.

To investigate the accumulation of some abiotic stress-related proteins during the course of both single and combined heat and drought treatments in *P. yunnanensis*, we performed protein immunoblot analysis with specific antibodies against plant mitogen-activated protein kinase 6 (MAPK6), heat shock protein 18.2 (HSP18.2), abscisic acid (ABA) synthase 9-cis-epoxycarotenoid dioxygenase (NCED), and dehydrin (Figure 7). Accumulations of these four proteins were induced to varying degrees by single high-temperature and drought stress treatments. Similar to the results of the 2-DE analysis, accumulations of proteins under high temperature stress were much larger than under drought stress (Figure 7). Under combined stress, however, these proteins showed different expressions. MAPK6 was induced from 0 to 24 h but inhibited after 24 h (Figure 7). Notably, the expression peak of NCED, which mediates the synthase of ABA, occurred 6 h after the start of the treatment (Figure 7). Unlike MPK6 and NCED, parallel changes occurred in the accumulation of HSP18.2 and dehydrin; they experienced sustained increases throughout the stress treatment (Figure 7).

## Discussion

### The effects of different treatments on *P. yunnanensis* plantlets

In plants, a series of integrated events at morphological, physiological, and proteomic levels are triggered by exposure to abiotic stresses [5]. Superoptimal temperatures can lead to changes in plant photosynthesis, protein synthesis, and cell contents [25]. As high temperatures cause strong evapotranspiration that induces drought, plants typically suffer from combined heat and drought rather than heat stress alone. Because environmental factors interact synergistically [26], [27] or antagonistically [26], plants can be influenced quite differently by a combination of stresses than by single factors. In the present study, we investigated the morphological and physiological changes of *P. yunnanensis* plantlets exposed for 48 h to high temperature, drought, and both stresses simultaneously. Our results consistently indicated that the plantlets were obviously influenced by sustained heat stress but barely affected by 48-h drought (Figure 1). Similar to the results of a previous study using the same species [18], we found that a combination of the two stresses directly damaged the plantlets and had an enhanced effect relative to the influence of either individual stress (Figure 1). To investigate potential plantlet response mechanisms, we further applied proteomics approaches to analyze the internal processes of plantlets treated by heat, drought, and combined heat and drought. The findings revealed by those analyses are discussed below.

### Chlorophyll fluorescence and photosynthesis-related proteins

The photosynthetic apparatus associated with PSII is highly sensitive to heat, drought, and various other stresses [13], [28] that usually reduce the photosynthetic rate and increase the rate of photorespiration [9], [29]. In the present study, a continuous reduction in photosynthetic rate, which reflected the level of stress, was deduced from the change in chlorophyll fluorescence (*F*_v_/*F*_m_) under three different stress regimes (Figure 2A and B). This result, which clearly reveals the serious impact of high temperature accompanied by drought on the photosynthetic system, was corroborated by a decline in ETRs (Figure 2). Three identified proteins related to photosynthesis – plasma membrane H+-ATPase (spot 13), oxygen-evolving enhancer protein 1 (spot 41), and phosphate import ATP-binding protein PstB (spot 93) – displayed distinctly reduced expression levels under combined heat and drought stress (Table S1). Under single heat or drought stress conditions, however, only the expression of phosphate import ATP-binding protein PstB was reduced significantly (Table S1). This result implies that a single stress, unlike combined stress, had a relatively minimal effect on plants and did not strongly interrupt the regulatory network of the photosynthetic system.

### Antioxidant enzymes and related proteins

ROS comprising H_2_O_2_, O_2_^−^, OH, and ^1^O_2_ are important signal molecules in plants [30]. Under normal conditions, ROS are maintained in homeostasis, with their excessive accumulation prevented by antioxidant enzymes and other substances located in different cell compartments. When plants are exposed to various stresses, ROS are typically induced in sufficient numbers to cause oxidative damage; as confirmed by several previous studies [29], 31,32, corresponding antioxidant molecules are induced in response. In the present study, we tested the accumulation of H_2_O_2_ and O_2_^−^ in conjunction with the activities of antioxidant enzymes (CAT, APX, SOD, and GR) and the expression of related proteins. Small amounts of H_2_O_2_ and O_2_^−^ were detected under individual heat and drought stress conditions (Figure 3C and D), and the activities of the four antioxidant enzymes increased significantly over the course of the stress treatments (Figure 4). When plants were subjected to combined stress, however, obviously different results were obtained. During the first 24 h, only small quantities of H_2_O_2_ and O_2_^−^ were induced (Figure 3C and D), with the antioxidant enzymes also stimulated (Figure 4). After 24 h of stress, ROS levels increased substantially (Figure 3C and D) while antioxidant enzyme activities gradually decreased (Figure 4), indicating that the plants' antioxidant systems may have been disrupted. Interestingly, proteomics analyses also revealed that several antioxidant proteins, namely 2-Cys peroxiredoxin (spot 1), putative ascorbate peroxidase APX5 (spot 9), catalase 2 (spot 14), glutathione S-transferase 16 (spot 25), and peroxidase 43 (spot 28), varied dramatically in expression level (Table S1) – a result generally consistent with observed changes in antioxidant enzyme activities. MDA, commonly used as an index of cellular oxidation levels [32], reflected the status of ROS equilibrium. In our study, the MDA content of *P. yunnanensis* plantlets gradually rose over the course of the different stress treatments, with the greatest increase recorded under combined stress (Figure 3B). Taken together, these results suggest that new equilibria were established under single heat or drought conditions to prevent oxidative damage. Under combined stress, a new equilibrium was also established during early stages (0–24 h); during late stages (24–48 h), however, severe oxidative damage occurred along with obvious phenotypic changes.

### Proline and proteins involved in abiotic stress

Proline, an osmotic regulator, can protect cells against heat and other stresses during various stages of acclimation [14]. Proline helps plants avoid oxidative damage and is considered to be an indicator of stress response at the cellular level in many plants [28]. Proline also has been suggested to mediate osmotic adjustment, stabilize macromolecules, serve as a compatible solute to protective enzymes, and store carbon and nitrogen for use during stress regimes such as heat and drought [33]. In our experiments, proline content rose gradually by various degrees under single heat and drought stress conditions, indicating its important role in stress response (Figure 3A). Under combined stress, in contrast, proline content initially increased but then declined (Figure 3A), implying the occurrence of two successive response phases. Similar evidence for these two phases came from the expression of NCED (Figure 7), a synthase of ABA, which is an important plant hormone modulating responses to abiotic stresses including heat, cold, and drought [34]. The various expressions of NCED indirectly suggest the significant roles and different regulatory functions of ABA during different stresses. Dehydrins are present in plants and can be induced by ABA, cold, salt, drought, and heat stress [35]. Western blotting revealed that dehydrin accumulated at different levels throughout the three different stress treatments (Figure 7), implying its significant role in resistance to these stresses.

Another peculiarity of plant response to abiotic stress is the abundant synthesis of sHSPs (17–30 kDa) [28], which constitute an important class of the HSP family. Members of the HSP family protect cells from the deleterious effects of extreme temperatures [14]. We identified three sHSPs –22-kDa (spot 12), 26.7-kDa (spot 16), and 23.1-kDa (spot 22) HSPs – using 2-DE as well as HSP18.2 detected by western blotting. All sHSPs detected under each stress, especially heat-related stress, gradually accumulated over the course of the treatment (Figure 7 and Table S1), indicating their important roles in stress resistance. Nevertheless, the protection conferred by sHSPs was not effective during later periods of combined stress.

Several other defense-related proteins, including desiccation-related protein LbLEA3_3–06 (spot 3), resistance protein (spot 6), late embryogenesis abundant protein D-113 (spot 18), and 3-isopropylmalate dehydratase small subunit (spot 82) were observed to be differentially expressed under different stress conditions (Table S1). Under single heat or drought stress conditions, these proteins were conformably up-regulated, indicating their roles in defense against these stresses (Table S1). Under combined stress, however, these proteins increased in early stages (0–12 or 24 h) and then declined (12 or 24–48 h) (Table S1). Changes in the expression of these abiotic stress-related proteins mirrored the physiological changes of the stress-treated plants.

### Proteins involved in cell and nucleic acid activities

The stability of cell and DNA activities is a reflection of the status of plant stress response, as well as the basis of defense against stress. Plant cells can change their structures and division activity to respond to harsh environmental conditions [29]. We observed several cell structural and division-related proteins that were differentially expressed during either individual or combined heat and drought stress conditions. For example, levels of small GTP-binding protein (spot 7) and ectoderm-neural cortex protein 2 (spot 48) decreased in response to the stress treatments (Table S1). Tetratricopeptide repeat protein 30A2 (spot 51) and leucine-rich PPR motif-containing protein (spot 63) exhibited different degrees of increase in *P. yunnanensis* plantlets exposed to different stresses (Table S1), whereas xyloglucan endotransglucosylase/hydrolase protein A (spot 30) was first induced but then decreased (Table S1). These results suggest that *P. yunnanensis* cell activities are actively regulated or passively influenced by high temperature and drought, similar to the reported response of *Portulaca oleracea* to high temperature conditions [29].

Although environmental stresses can cause nucleic acid damage, many preventative and damage-repair mechanisms exist to enable plant survival [36]–[38]. During heat or combined heat and drought stress, a putative retrotransposon protein (spot 8) and arginine-tRNA ligase (spot 67) were mainly decreased while nucleotide-binding protein Sala_2050 (spot 76) was mainly up-regulated; a positive correlation was observed between expression level and degree of stress (Table S1). However, the protein designated as single-stranded DNA-binding protein (spot 84) was induced by heat stress but decreased under combined stress (Table S1), supporting the tentative conclusion that the effects of heat and drought stress are exacerbated when the two stresses are combined. These results indicate that various related proteins, despite some degree of down-regulation, work in concert to maintain normal nucleic acid metabolism under stress.

### Proteins involved in energy metabolism

On the basis of several proteomics analyses, proteins related to energy metabolism have been proven to play an important role in plant response to abiotic stress. Yang et al. [29] reported that several material- and energy-associated proteins in the thermotolerant plant *Portulaca oleracea* increase in response to high temperature stress, thereby contributing to its heat tolerance. Li et al. [32] found that the expression of two ATP synthases in *Kobresia pygmaea* were up-regulated along an elevational gradient corresponding to increasingly harsh environmental conditions. Because photosynthesis is greatly suppressed under stress, respiration, which is less susceptible and more adaptive than photosynthesis, can become a determinative factor for plant survival [39] by meeting the increased demand for ATP. In the present study, we obtained results in agreement with previous studies. One of two mitochondrial proteins of the ATP synthase beta subunit (spots 44 and 65) was increased under high temperature stress despite showing no significant difference under drought conditions (Table S1). Three homologs (spots 19, 24, and 29) of the large subunit of ribulose-1,5-bisphosphate carboxylase/oxygenase, which is involved in carbon assimilation and photorespiration [40], were differentially up-regulated under individual drought or heat stress (Table S1), indicating their stress-response contributions. Stronger induction of a greater number of proteins related to energy metabolism was observed during early stages (0–24 h) of the combined stress treatment. Except for one protein whose expression remained at a high level, these proteins were then differentially decreased during later stages (24–24 h) (Table S1). These results support our conclusion that excessive exposure to heat and drought obstructs the defense system of *P. yunnanensis* plantlets.

### Different types of protein enzymes

Protein enzymes, which primarily function as biological catalysts, participate in various plant life activities. Our proteomics analyses revealed that many enzymes besides antioxidant enzymes, including synthases, kinases, phosphatases, transferases and mutases, vary dramatically in expression levels under different stresses, with several specific changes observed under combined stress (Table S1). The observed expression changes suggest the importance or sensitivity of these enzymes in stress response. In particular, the basic post-translation protein modifications of phosphorylation and dephosphorylation modulate plant response to environmental stress [41]. We identified four protein kinases – PTI1-like tyrosine-protein kinase At3g15890 (spot 35), protein kinase C-like 1B (spot 74), acetate kinase (spot 81), and acetylglutamate kinase (spot 92) – and two phosphatases – probable protein phosphatase 2C 15 (spot 11) and phytochrome-associated serine/threonine protein phosphatase 1 (spot 26). Most of these enzymes were differentially up-regulated during different stresses until the combined stresses exceeded plant tolerance limits (after 24 h) (Table S1). These results suggest that the enzymes enhance plant response to single heat or drought stress and early stages of combined stress.

## Conclusions

**Figure 8:**
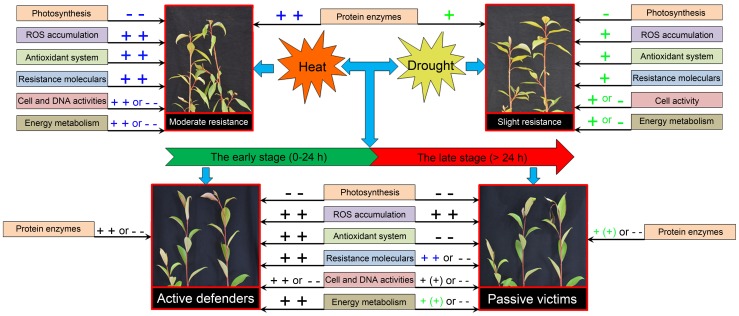
Schematic illustration of a proposed model for the process of *Populus yunnanensis* plantlet response to high temperature, drought, and a combination of the two stresses. The symbols “+” and “−” represent slight increases and decreases, respectively, while “+ +” and “− −” represent substantial changes. Information in parentheses is optional. Green, blue, and black symbols are used to show gradual increases in the amounts of proteins and substances involved in the process.

In this study, we performed comparative physiological and proteomic profiling to investigate how plantlets of poplar (*P. yunnanensis*), a common broadleaved deciduous tree of southwestern China, respond to extreme high temperature accompanied by drought, with individual treatments of heat and drought used for comparison. Our results provide insight into how woody plants may respond to excessive heat, as is expected with global warming. When exposed to individual heat or drought stress, plantlets exhibited different levels of resistance, similar to results reported from many previous studies [7]–[9]. Nevertheless, as indicated in our proposed model (Figure 8), we detected two stages of response to combined stress. During the first stage, between 0 and 24 h, plants actively defended themselves to establish a new cellular homeostasis through both physiological and proteomic responses. This activity explains why plant morphology during this period barely changed. During the second stage, plants were overwhelmed by stress. ROS homeostasis was defeated by ROS overproduction, antioxidant enzyme activities declined, and the synthesis of some protective substances, such as proline and ABA, was suppressed. As a result, photosynthesis decreased sharply, and buds began to die despite continued accumulation of sHSPs and dehydrin. Our results indicate that extreme heat may threaten some non-resistant plants. Plant stress tolerance may be related to plant age [33], with differences existing between young plants and adults. Although excessive heat may not impact adult individuals, it can reduce population density and community structure by killing young plants. As a consequence, seedling fates are worthy of attention. At the same time, many previous studies have revealed significant sexual differences in abiotic stress responses in *P. yunnanensis*, with females usually experiencing greater negative effects than males [16]–[23]. This observation suggests that female plantlets may be more seriously damaged by exposure to combined heat and drought stress, thus requiring more attention during extreme conditions.

## Materials and Methods

### Ethics statement

Plant materials used in this study were collected from the Kunming suburbs (E 102°44′24″, N 25°8′20″), Yunnan Province, China. No specific collecting permits were required for this location, as it was located adjacent to our institute, the Kunming Institute of Botany of the Chinese Academy of Sciences. Plant administration was under the auspices of the Kunming City Forestry Bureau, with any studies beneficial or non-damaging to plants permitted and supported by relevant departments. We confirm that the plant we used is a common native species that is neither endangered nor protected.

### Plant materials and treatment

Yunnan poplar cuttings were obtained from male plants in March 2012. After survival in the field for 30 d, 150 healthy plantlets with an average of five nodes and a height of 15–20 cm were transplanted into plastic pots (15 cm ×20 cm) containing equal biomass in a greenhouse. The plants were grown in the greenhouse for 30 d under sunlight conditions (23–25°C day and 18–20°C night) and watered daily on a regular schedule with 100 ml of water per pot. The plantlets were then divided into three groups and subjected to different stresses in an incubator with a 12-h photoperiod (800 µmol photons m^−2^ s^−1^ light intensity). One of three stress treatments was applied to each group: (1) a constant temperature of 40°C with regular watering, (2) the normal pre-treatment temperature regime with no watering, or (3) a constant temperature of 40°C with no watering. Treatments were begun simultaneously during the day time, prior to the scheduled daily watering, with treatment continuing for 0, 6, 12, 24, or 48 h. After plant morphological changes were recorded, the fourth to sixth leaves from the top were harvested to determine their physiological and biochemical properties for each treatment. Five replicates were performed per experiment, and samples from the 0-h treatment were used as controls for the data analysis.

### Leaf-change observations and detection

Leaves showing obvious necrotic lesions and crinkling were considered to be withered. Before leaf harvesting, the number of withered leaves was recorded at each time point for all treatments. To measure water content of leaves subjected to stresses, the fourth to sixth leaves from the bottom were collected and any surface impurities removed. Fresh weights (FWs) were measured, with dry weights (DWs) recorded after drying at 80°C for 48 h [42].

### Analysis of chlorophyll fluorescence

Chlorophyll fluorescence was analyzed as previously described [29], [31] with a pulse-amplitude modulated chlorophyll fluorometer (Heinz Walz GmbH, Effeltrich, Germany). Briefly, *P. yunnanensis* plantlets after treatment were dark-adapted for 30 min to measure the maximal quantum yield of PSII (*F*_v_ */F*_m_), which was determined for each sample by analyzing a whole leaf. The maximal fluorescence (*F*_m_) was recorded using a 0.8-s pulsed light at 4,000 µmol s^−1^ m^−2^, and minimal fluorescence (*F*_o_) was recorded during the weak measuring pulses. ETRs at a given actinic irradiance were calculated according to the instrument manual as follows: (*F*_m_' – *F*_s_)/*F*_m_' × PAR ×0.5×α, where (*F*_m_' − *F*_s_)/*F*_m_' is the quantum yield of PSII (φPSII) in light, PAR is the photosynthetically active irradiance, 0.5 is the assumed proportion of absorbed quanta used by PSII reaction centers, and α is the absorbance for poplar leaves.

### Proline and MDA content measurements

Proline content was measured as previously reported [43]. Approximately 0.5 g of fresh leaves of each sample was homogenized in 8 ml of 3% aqueous sulfosalicylic acid, and the homogenate was centrifuged at 2,000×*g* for 10 min. Two milliliters each of the extract, acidic ninhydrin, and glacial acetic acid were heated for 1 h in a boiling water bath, with the reaction then terminated in an ice bath. The reaction mixture was extracted with 4 ml toluene, with vigorous mixing using a test-tube stirrer for 15–20 s. The chromophore-containing toluene was aspirated from the aqueous phase and warmed to room temperature, and its absorbance was read at 520 nm using toluene for a blank. The proline concentration was determined from a standard curve and calculated on a DW basis as follows:

(µg ml^−1^ proline × ml toluene) ×5 (g sample)^−1^ =  µg proline g^−1^ DW material.

MDA content was determined as described previously [44]. Approximately 0.5determined from a standard curve and g of fresh leaves per sample was homogenized in 10determined from a standard curve and ml of 10% trichloroacetic acid (TCA) and centrifuged at 12,000×*g* for 10determined from a standard curve and min. Two milliliters of 0.6% thiobarbituric acid in 10% TCA was then added to an aliquot of 2determined from a standard curve and ml of the supernatant. The mixture was heated in boiling water for 30determined from a standard curve and min and then quickly cooled in an ice bath. After centrifugation at 10 000×*g* for 10determined from a standard curve and min, the absorbance of the supernatant at 450, 532, and 600determined from a standard curve and nm was determined. The MDA concentration, which was expressed as nmol g^−1^ DW, was estimated from the formula: C (nmol ml^−1^)  = 6.45 (A_532_−A_600_) −0.56A_450_.

### *In situ* H_2_O_2_ and O_2_^−^ detection

*In situ* detection of H_2_O_2_ and O_2_^−^ were performed using a previously reported method with some modifications [45]. To detect H_2_O_2_, three leaf discs drilled at specific time points during different treatments were vacuum-infiltrated in 10 ml of 1 mg ml^−1^ diaminobenzidine solution for 2 h, and were then cleared in boiling ethanol (95%) for 10 min. The samples were subsequently stored and examined in 95% ethanol. The amount of O_2_^−^ in leaves was monitored by 10^−2^ M nitro-blue tetrazolium (NBT) reduction at specific time points. Three leaf pieces were vacuum-infiltrated with 10 ml NBT for 2 h, cleared in boiling ethanol (95%) for 10 min, and stored and examined in 95% ethanol.

### Antioxidant enzyme activity assays

Approximately 0.5 g of leaves from each sample was homogenized in 10 ml extraction buffer (50 mM sodium phosphate [pH 7.0], 1 mM EDTA, 1 mM dithiothreitol [DTT], 1 mM glutathione, 5 mM MgCl_2_·6H_2_O, 1% [w/v] PVP-40, and 20% [v/v] glycerin). The homogenates were centrifuged at 12,000×*g* for 15 min at 4°C, and the total soluble protein content of the supernatants was measured by the Bradford method [46]. CAT (EC1.11.1.6), APX (EC1.11.1.11), SOD (EC1.15.1.1) and GR (EC1.8.1.7) activities were determined as previously described [47], [48].

### Protein extraction and 2-DE

Protein extraction and 2-DE were performed as reported previously [49], with some modifications. Approximately 10–20 g of leaves from samples exposed to different treatments for 0, 6, 12, 24, or 48 h were ground in liquid nitrogen, and the total soluble proteins were extracted on ice in acetone containing 10% (w/v) TCA and 0.07% (w/v) DTT. The homogenates were held at −20°C for 4 h and then centrifuged at 8,000×*g* for 30 min at 4°C. The pellets were washed with acetone containing 0.07% (w/v) DTT at −20°C for 30 min and then centrifuged at 8,000×*g* for 20 min at 4°C; this step was performed a total of three times. Finally, the pellets were vacuum-dried and then dissolved in lysate (7 M urea, 2 M thiourea, 4% [w/v] CHAPS, and 60 mM DTT) for 2 h at room temperature with intermittent shocking, followed by centrifugation at 12,000×*g* for 20 min at 20°C. The supernatants were collected for the 2-DE experiments, which were performed in triplicate.

Extracted proteins (1,200 µg) were first separated by isoelectric focusing (IEF) using gel strips to build an immobilized non-linear pH gradient from 4 to 7 (Immobiline Dry Strip, pH 4–7 NL, 17 cm; Bio-Rad, Hercules, CA, USA) and then by sodium dodecyl sulfate-polyacrylamide gel electrophoresis (SDS-PAGE) using 12.5% polyacrylamide. The strips were rehydrated for 14 h in 320 µl of dehydration buffer and then focused at 20°C for a total of 64 kV-h with a PROTEAN IEF Cell system (Bio-Rad). After IEF, the strips were equilibrated for 20 min, first in equilibration buffer I (6 M urea, 0.375 M Tris [pH 8.8], 2% [w/v] SDS, 20% [v/v] glycerol, and 2% [w/v] DTT) and then in equilibration buffer II (6 M urea, 0.375 M Tris [pH 8.8], 2% [w/v] SDS, 20% [v/v] glycerol, and 2% [w/v] iodoacetamide). The equilibrated strips were placed over 12.5% (w/v) SDS-PAGE gels for 2-DE at 25 mA for 5 h. Gels were stained with colloidal CBB. After staining, gels were scanned using PDQuest 2D analysis software (Bio-Rad) on the basis of their relative volumes as described by Bai et al. [31]. To compensate for subtle differences in sample loading or gel staining/destaining during individual experiments, the volume of each spot was normalized [50].

### Spot digestion and protein identification for MS analyses

Protein spots displaying significant changes in abundance following plant exposure to heat, drought, or their combination were excised manually from colloidal CBB-stained 2-DE gels using sterile pipette tips. Spots were transferred to 1.5-ml sterile tubes, destained with 50 mM NH_4_HCO_3_ for 1 h at 40°C, reduced with 10 mM DTT in 100 mM NH_4_HCO_3_ for 1 h at 60°C, and incubated with 40 mM iodoacetamide in 100 mM NH_4_HCO_3_ for 30 min. Gels were then minced, air-dried, and rehydrated in 12.5 ng µl^−1^ sequencing-grade modified trypsin (Promega, Fitchburg, WI, USA) in 25 mM NH_4_HCO_3_ overnight at 37°C. Tryptic peptides were extracted three times from the gel grains using 0.1% trifluoroacetic acid (TFA) in 50% acetonitrile. Supernatants were concentrated to approximately 10 µl using a SpeedVac (Thermo Fisher, Waltham, MA, USA) and then desalted using reversed-phase ZipTip pipette tips (C18, P10; Millipore, Billerica, MA, USA). Peptides were eluted with 50% acetonitrile and 0.1% TFA. Protein spots that differed in concentration by more than 1.5-fold and differed significantly (Student's *t*-test, *P*<0.05) compared with the control were analyzed by MS.

Lyophilized peptide samples were dissolved in 0.1% TFA, and MS analysis was conducted using a 4800 Plus MALDI-TOF/TOF Proteomics Analyzer (Applied Biosystems, Foster City, CA, USA). MS acquisition and processing parameters were set to reflector-positive mode and an 800–3,500-Da acquisition mass range, respectively. The laser frequency was 50 Hz, and each sample spectrum was acquired over 700 laser pulses. For secondary MS analysis, four to six ion peaks with signal-to-noise ratios exceeding 100 were selected from each sample as precursors. TOF/TOF signal data for each precursor were then accumulated from 2,000 laser pulses. Primary and secondary mass spectra were transferred to Excel files and compared against a non-redundant NCBI protein database (NCBI-nr 20101014) restricted to Viridiplantae (i.e., green plants) using the MASCOT search engine (www.matrixscience.com). The following search parameters were used: no molecular weight restriction, one missed trypsin cleavage allowed, iodoacetamide-treated cysteine, oxidation of methionine, a peptide tolerance of 100 ppm, and an MS/MS tolerance of 0.25 Da. Protein identifications were validated manually based on at least three matching peptides. Keratin contamination was removed, and the MOWSE threshold was set above 20 (*P*<0.05). Only significant hits in the MASCOT probability analysis were accepted as protein identifications.

### Western blotting

SDS-PAGE was performed as described previously [51] using 12% (w/v) polyacrylamide slab gels. For western blot analysis, the protein samples were electroblotted onto polyvinylidene difluoride membranes using a Trans-Blot cell (Bio-Rad). After transfer, the membranes were probed with the appropriate primary antibodies and HRP-conjugated goat anti-rabbit secondary antibody (Promega), and the signals were detected using an ECL kit (GE, Evansville, IN, USA). The primary antibodies were diluted as follows: polyclonal antibody against MAPK6 (1∶1,000), HSP18.2 (1∶2,000), NCED (1∶3,000), dehydrin (1∶3,000), and actin (1:2,000).

### Statistical analysis

Statistical analyses were performed using SPSS version 12.0. ANOVA for all variables from measurements were used for testing the treatment differences. Differences were considered significant at the *P*<0.05 level.

## Supporting Information

Figure S1 **Representative set of 2-D gels of samples subjected to high temperature stress.** Marked numbers represent differentially expressed proteins in the treatment.
(TIF)

Figure S2 **Representative set of 2-D gels of samples subjected to drought stress.** Marked numbers represent differentially expressed proteins in the treatment.
(TIF)

Figure S3 **Representative set of 2-D gels of samples subjected to a combination of high temperature and drought.** Marked numbers represent differentially expressed proteins in the treatment.
(TIF)

Table S1 **Protein spot intensity ratios from different treatments at different treatment times (6, 12, 24, and 48 h) relative to the control (0 h).**
(DOCX)
