# Supplementary figures and images for: Comparative Physiological and Proteomic Analyses of Poplar (Populus yunnanensis) Plantlets Exposed to High Temperature and Drought
Source: PLoS One. 2014 Sep 16;9(9):e107605. doi: 10.1371/journal.pone.0107605 (PMC4167240; doi:10.1371/journal.pone.0107605)

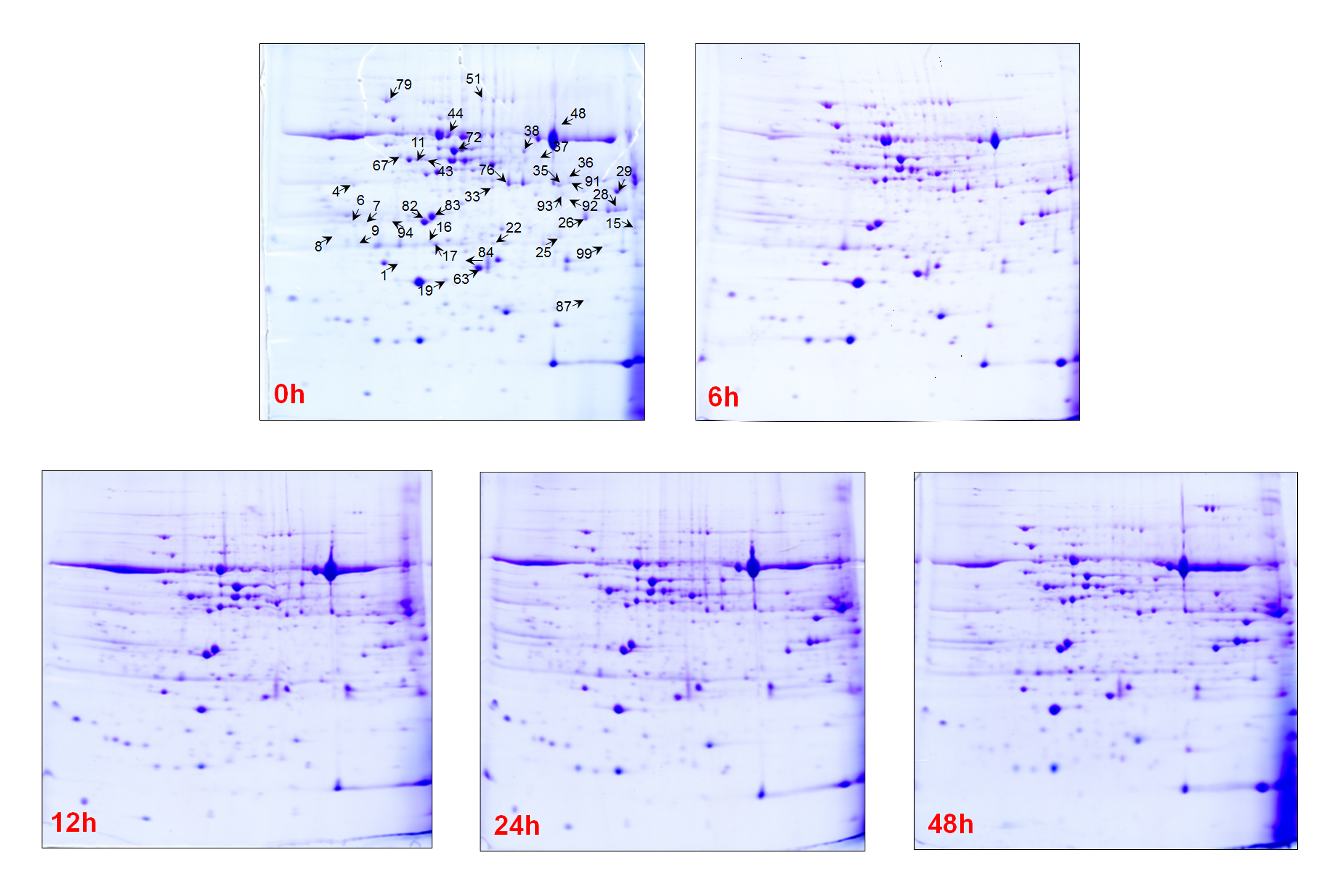

Supplement: Figure S1 — Representative set of 2-D gels of samples subjected to high temperature stress. Marked numbers represent differentially expressed proteins in the treatment. (TIF) [file pone.0107605.s001.tif]

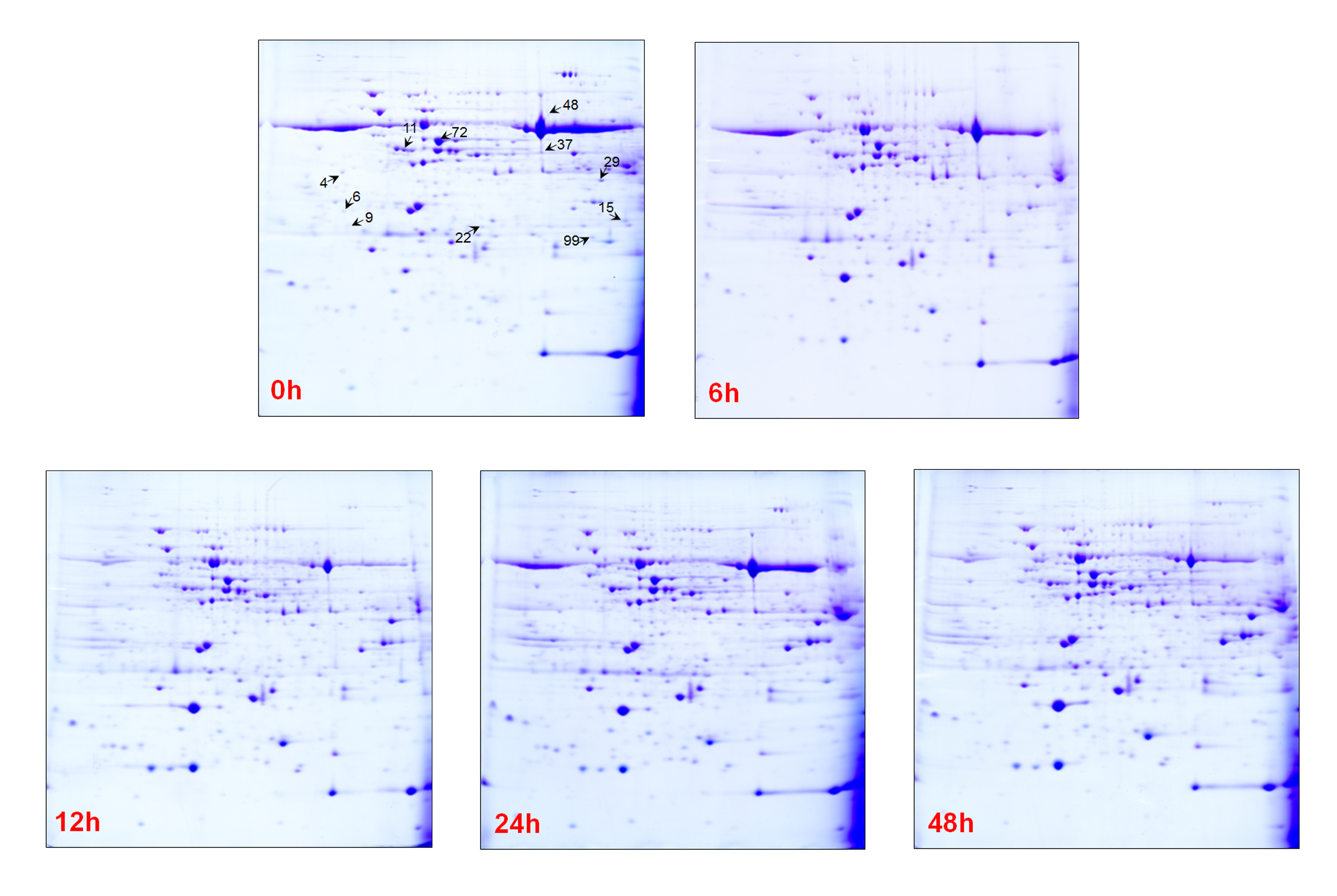

Supplement: Figure S2 — Representative set of 2-D gels of samples subjected to drought stress. Marked numbers represent differentially expressed proteins in the treatment. (TIF) [file pone.0107605.s002.tif]

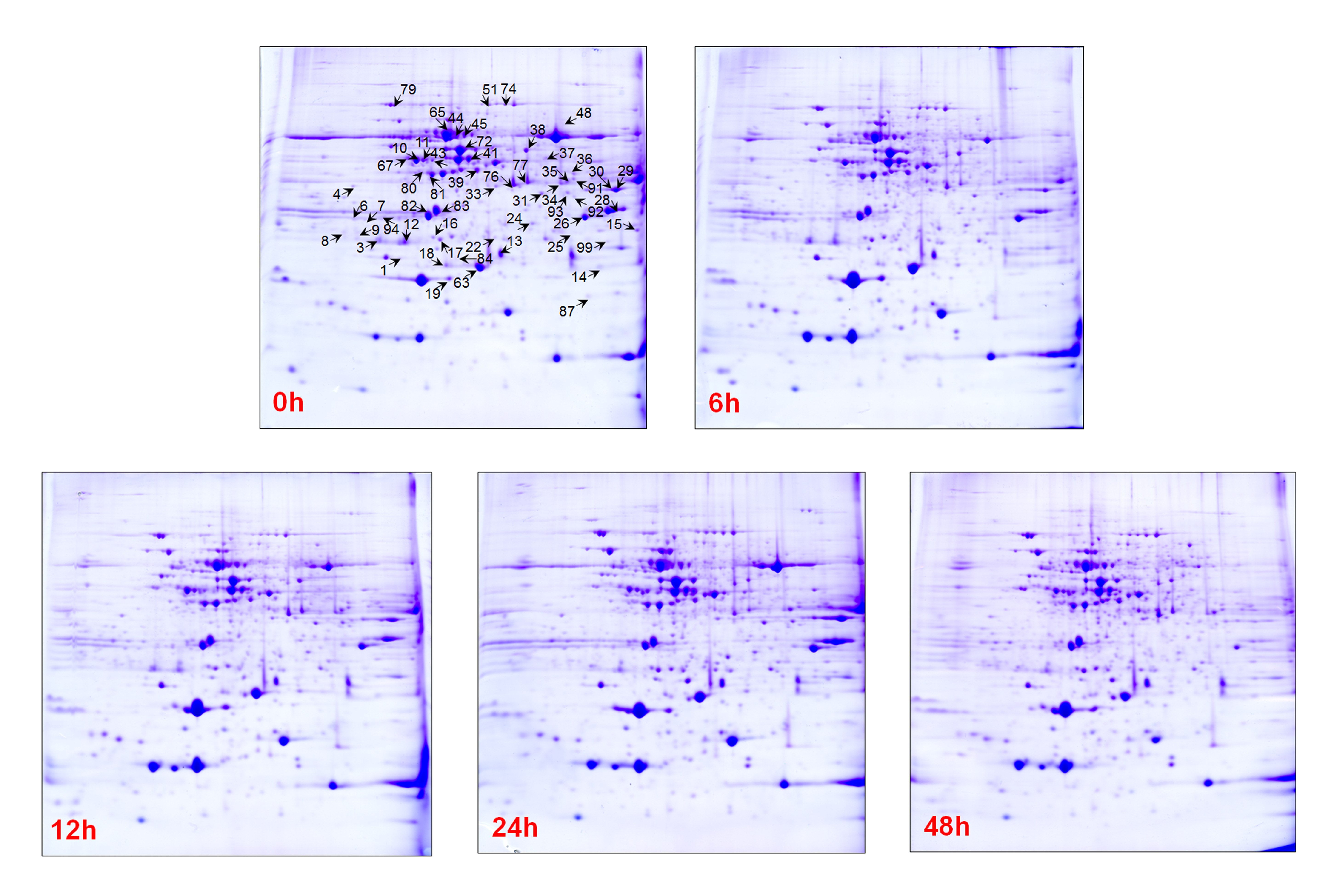

Supplement: Figure S3 — Representative set of 2-D gels of samples subjected to a combination of high temperature and drought. Marked numbers represent differentially expressed proteins in the treatment. (TIF) [file pone.0107605.s003.tif]
