# Supplementary material for: Comparative Physiological and Proteomic Analyses of Poplar (Populus yunnanensis) Plantlets Exposed to High Temperature and Drought
Source: PLoS One. 2014 Sep 16;9(9):e107605. doi: 10.1371/journal.pone.0107605 (PMC4167240; doi:10.1371/journal.pone.0107605)
Supplement: Table S1 — Protein spot intensity ratios from different treatments at different treatment times (6, 12, 24, and 48 h) relative to the control (0 h). (DOCX) [file pone.0107605.s004.docx]

**Table S1**

| Spot | Ratio | | | | | | | | | | | | | | |
| --- | --- | --- | --- | --- | --- | --- | --- | --- | --- | --- | --- | --- | --- | --- | --- |
|  |  | High temperature | | | |  | Drought | | | |  | High temperature and drought | | | |
|  |  | 6h/0h | 12h/0h | 24h/0h | 48h/0h |  | 6h/0h | 12h/0h | 24h/0h | 48h/0h |  | 6h/0h | 12h/0h | 24h/0h | 48h/0h |
| 1 |  | 1.11 | 1.28 | 1.47 | 1.69 |  | 1.11 | 1.19 | 1.24 | 1.32 |  | 1.58 | 1.74 | 1.93 | 0.90 |
| 3 |  | 1.04 | 1.19 | 1.34 | 1.41 |  | 1.08 | 1.13 | 1.16 | 1.22 |  | 1.08 | 1.25 | 0.41 | 0.37 |
| 4 |  | 1.36 | 1.45 | 1.64 | 1.78 |  | 1.08 | 1.17 | 1.37 | 1.51 |  | 1.63 | 1.73 | 1.92 | 1.78 |
| 6 |  | 1.89 | 2.53 | 2.29 | 3.04 |  | 1.30 | 1.64 | 1.70 | 1.86 |  | 2.78 | 2.83 | 3.12 | 2.16 |
| 7 |  | 0.61 | 0.83 | 1.00 | 1.49 |  | 1.07 | 1.02 | 1.00 | 0.92 |  | 0.81 | 0.64 | 0.81 | 0.34 |
| 8 |  | 0.98 | 0.81 | 0.81 | 0.60 |  | 0.98 | 0.88 | 1.15 | 1.29 |  | 0.73 | 0.60 | 0.34 | 0.19 |
| 9 |  | 1.06 | 2.04 | 2.14 | 2.30 |  | 1.06 | 1.23 | 1.33 | 1.76 |  | 1.06 | 3.94 | 4.58 | 0.41 |
| 10 |  | 0.97 | 1.03 | 0.85 | 0.77 |  | 1.01 | 1.03 | 1.04 | 1.15 |  | 0.95 | 0.90 | 0.84 | 0.66 |
| 11 |  | 1.39 | 1.66 | 1.78 | 2.09 |  | 1.06 | 1.15 | 1.35 | 1.59 |  | 1.31 | 1.57 | 1.69 | 2.18 |
| 12 |  | 1.03 | 1.13 | 1.27 | 1.33 |  | 1.02 | 1.04 | 1.06 | 1.10 |  | 1.06 | 1.16 | 1.29 | 1.55 |
| 13 |  | 0.96 | 0.95 | 0.87 | 0.78 |  | 0.98 | 0.97 | 0.91 | 0.89 |  | 0.94 | 0.87 | 0.79 | 0.46 |
| 14 |  | 1.02 | 1.14 | 1.19 | 1.21 |  | 1.02 | 1.04 | 1.09 | 1.13 |  | 1.15 | 1.20 | 1.50 | 1.03 |
| 15 |  | 1.08 | 0.99 | 0.74 | 0.58 |  | 0.84 | 0.74 | 0.68 | 0.58 |  | 0.78 | 0.50 | 0.43 | 0.34 |
| 16 |  | 1.39 | 1.71 | 1.81 | 2.00 |  | 1.03 | 1.18 | 1.28 | 1.47 |  | 1.57 | 1.89 | 1.99 | 2.36 |
| 17 |  | 1.18 | 1.25 | 1.76 | 1.88 |  | 1.02 | 1.08 | 1.19 | 1.31 |  | 1.28 | 1.34 | 2.24 | 1.88 |
| 18 |  | 1.01 | 1.06 | 1.10 | 1.15 |  | 1.00 | 1.02 | 1.04 | 1.10 |  | 1.03 | 1.06 | 1.10 | 0.56 |
| 19 |  | 1.31 | 1.64 | 1.84 | 1.88 |  | 1.10 | 1.11 | 1.31 | 1.35 |  | 2.69 | 2.17 | 2.37 | 1.88 |
| 22 |  | 1.78 | 2.20 | 3.57 | 3.89 |  | 1.34 | 1.76 | 2.25 | 2.57 |  | 2.21 | 2.64 | 4.01 | 4.33 |
| 24 |  | 1.36 | 1.29 | 1.11 | 1.35 |  | 1.05 | 1.14 | 1.11 | 1.05 |  | 1.51 | 1.67 | 1.42 | 0.89 |
| 25 |  | 1.04 | 1.18 | 1.27 | 1.76 |  | 1.01 | 1.08 | 1.11 | 1.17 |  | 1.11 | 1.52 | 1.65 | 0.91 |
| 26 |  | 1.08 | 1.15 | 1.07 | 1.58 |  | 1.04 | 1.08 | 1.11 | 1.14 |  | 1.11 | 1.19 | 1.57 | 0.48 |
| 28 |  | 1.19 | 1.31 | 1.64 | 1.92 |  | 1.19 | 1.31 | 1.33 | 1.38 |  | 1.96 | 2.07 | 2.10 | 0.84 |
| 29 |  | 1.09 | 1.16 | 2.08 | 2.38 |  | 1.09 | 1.12 | 1.17 | 1.50 |  | 1.18 | 1.21 | 2.05 | 2.91 |
| 30 |  | 1.35 | 0.87 | 0.83 | 0.74 |  | 1.07 | 0.96 | 0.92 | 0.84 |  | 1.35 | 0.33 | 0.34 | 1.19 |
| 31 |  | 1.17 | 1.35 | 0.87 | 1.02 |  | 1.03 | 1.07 | 1.12 | 1.04 |  | 1.31 | 1.49 | 1.58 | 0.73 |
| 33 |  | 1.23 | 1.11 | 1.92 | 1.61 |  | 1.07 | 1.11 | 1.61 | 1.46 |  | 1.39 | 0.96 | 2.40 | 1.46 |
| 34 |  | 0.95 | 0.86 | 1.03 | 0.84 |  | 0.97 | 0.96 | 1.02 | 0.94 |  | 0.36 | 0.86 | 1.13 | 0.74 |
| 35 |  | 0.46 | 0.79 | 1.05 | 1.06 |  | 0.87 | 0.84 | 0.87 | 0.95 |  | 0.46 | 0.79 | 1.05 | 1.06 |
| 36 |  | 0.83 | 0.58 | 1.01 | 0.95 |  | 1.00 | 0.92 | 0.84 | 0.73 |  | 0.66 | 0.75 | 0.84 | 0.78 |
| 37 |  | 1.32 | 1.88 | 2.32 | 1.65 |  | 1.16 | 1.39 | 1.66 | 1.82 |  | 2.48 | 3.70 | 0.66 | 1.65 |
| 38 |  | 1.14 | 1.71 | 1.67 | 1.42 |  | 1.14 | 1.36 | 1.41 | 1.42 |  | 1.14 | 2.06 | 1.15 | 0.55 |
| 39 |  | 1.11 | 0.90 | 1.19 | 0.92 |  | 1.05 | 0.96 | 1.07 | 0.98 |  | 1.41 | 0.73 | 1.78 | 1.39 |
| 41 |  | 1.22 | 1.04 | 1.19 | 1.27 |  | 1.07 | 1.08 | 1.12 | 1.20 |  | 0.75 | 0.65 | 0.49 | 0.34 |
| 43 |  | 1.23 | 1.25 | 1.40 | 1.54 |  | 1.06 | 1.08 | 1.15 | 1.28 |  | 1.32 | 1.33 | 1.74 | 1.96 |
| 44 |  | 1.13 | 1.22 | 1.36 | 1.53 |  | 1.04 | 1.09 | 1.23 | 1.27 |  | 1.17 | 1.22 | 1.36 | 0.93 |
| 45 |  | 1.02 | 1.22 | 1.43 | 1.29 |  | 1.02 | 1.07 | 1.14 | 1.22 |  | 1.02 | 1.43 | 1.57 | 0.94 |
| 48 |  | 0.83 | 0.79 | 0.65 | 0.56 |  | 0.93 | 0.89 | 0.75 | 0.66 |  | 0.53 | 0.49 | 0.45 | 0.36 |
| 51 |  | 1.72 | 0.91 | 1.47 | 1.92 |  | 1.15 | 1.20 | 1.33 | 1.49 |  | 1.72 | 0.91 | 1.47 | 1.92 |
| 63 |  | 1.48 | 1.40 | 2.35 | 1.58 |  | 1.15 | 1.29 | 1.38 | 1.47 |  | 1.48 | 1.40 | 2.35 | 1.58 |
| 65 |  | 1.19 | 1.30 | 1.45 | 1.48 |  | 1.00 | 1.05 | 1.15 | 1.21 |  | 1.19 | 1.46 | 1.77 | 0.54 |
| 67 |  | 0.67 | 0.66 | 0.72 | 0.28 |  | 0.98 | 0.93 | 0.95 | 0.67 |  | 0.67 | 0.66 | 0.72 | 0.28 |
| 72 |  | 0.39 | 0.18 | 0.23 | 1.06 |  | 0.87 | 0.66 | 0.71 | 0.77 |  | 0.39 | 0.18 | 0.23 | 1.06 |
| 74 |  | 1.13 | 1.18 | 1.26 | 1.47 |  | 1.10 | 1.12 | 1.14 | 1.23 |  | 1.13 | 1.22 | 1.63 | 1.71 |
| 76 |  | 1.08 | 1.51 | 1.27 | 1.18 |  | 1.03 | 1.16 | 1.27 | 1.18 |  | 2.09 | 2.02 | 1.27 | 1.18 |
| 77 |  | 1.15 | 1.42 | 1.42 | 1.15 |  | 1.08 | 1.21 | 1.27 | 1.15 |  | 1.51 | 2.14 | 2.14 | 1.00 |
| 79 |  | 2.16 | 1.73 | 1.43 | 1.20 |  | 1.31 | 0.87 | 1.00 | 1.03 |  | 4.74 | 2.58 | 2.29 | 2.41 |
| 80 |  | 0.82 | 0.94 | 0.75 | 0.92 |  | 0.97 | 0.94 | 0.89 | 0.78 |  | 0.75 | 1.02 | 0.60 | 1.06 |
| 81 |  | 1.08 | 1.16 | 1.23 | 1.09 |  | 1.02 | 1.04 | 1.08 | 1.12 |  | 1.21 | 1.26 | 1.54 | 0.33 |
| 82 |  | 1.21 | 1.24 | 1.31 | 0.15 |  | 1.04 | 1.06 | 1.07 | 1.20 |  | 1.16 | 1.82 | 1.96 | 1.51 |
| 83 |  | 0.92 | 0.74 | 0.67 | 0.52 |  | 0.96 | 0.89 | 0.82 | 0.76 |  | 0.20 | 0.26 | 0.29 | 0.25 |
| 84 |  | 1.08 | 1.19 | 1.95 | 0.94 |  | 1.04 | 1.10 | 1.19 | 1.04 |  | 0.98 | 0.91 | 3.84 | 0.94 |
| 87 |  | 2.17 | 0.32 | 0.92 | 1.75 |  | 1.27 | 1.29 | 1.22 | 1.44 |  | 2.31 | 0.34 | 0.97 | 1.86 |
| 91 |  | 1.07 | 1.22 | 1.37 | 1.66 |  | 1.07 | 1.11 | 1.19 | 1.29 |  | 1.26 | 1.33 | 1.52 | 2.03 |
| 92 |  | 1.17 | 1.37 | 1.59 | 1.84 |  | 1.03 | 1.10 | 1.21 | 1.30 |  | 1.44 | 1.91 | 2.40 | 0.76 |
| 93 |  | 0.90 | 0.75 | 0.71 | 0.64 |  | 0.98 | 0.90 | 0.87 | 0.83 |  | 0.75 | 0.70 | 0.66 | 0.26 |
| 94 |  | 0.75 | 0.66 | 0.61 | 0.58 |  | 0.96 | 0.87 | 0.83 | 0.72 |  | 0.53 | 0.31 | 0.47 | 0.58 |
| 99 |  | 1.47 | 1.62 | 3.30 | 4.64 |  | 1.25 | 1.38 | 1.69 | 2.24 |  | 1.97 | 2.12 | 4.80 | 6.65 |
